# Inverse association between dietary fiber intake and gallstone disease in U.S. adults: a cross-sectional study from the NHANES database

**DOI:** 10.3389/fnut.2025.1624173

**Published:** 2025-07-01

**Authors:** Dianbao Zuo, Ming Sang, Xiaodong Sun, Guoping Chen, Kangkang Ji

**Affiliations:** ^1^Research Center for Translational Medicine, Hubei Key Laboratory of Wudang Local Chinese Medicine Research, Hubei Provincial Clinical Research Center for Parkinson’s Disease at Xiangyang No.1 People’s Hospital, Hubei University of Medicine, Xiangyang, China; ^2^College of Biomedicine and Health, Huazhong Agricultural University, Wuhan, Hubei, China; ^3^Department of Clinical Medical Research, Binhai County People’s Hospital, Clinical Medical College of Yangzhou University, Yancheng, Jiangsu, China

**Keywords:** dietary fiber, gallstone, cross-sectional, NHANES, dose-response

## Abstract

**Background:**

The cross-sectional association of dietary fiber intake with gallstone disease in United States adults remains to be comprehensively elucidated.

**Methods:**

We used the National Health and Nutrition Examination Survey (NHANES) data from 2017–2023. The assessment of dietary fiber intake was derived from 24-h dietary recalls. Stratified analyses were then used to demonstrate dietary fiber intake corresponding to different groups of gallstone and non-gallstone conditions. The use of weighted logistic regression was employed to explore the correlation between dietary fiber intake and gallstone disease. Subgroup and interaction analyses were used to identify potential interacting factors. Additionally, restricted cubic spline was used to assess the dose–response between dietary fiber and gallstone risk.

**Results:**

The study population comprised 9,273 patients, with a gallstone prevalence of 10.47% (971 cases). In the context of various subgroups, patients diagnosed with gallstones exhibited a reduced dietary fiber intake in comparison with individuals not bearing gallstones. In the fully adjusted model, an inverse association was observed between dietary fiber intake and gallstone disease (odds ratio (OR), 95% confidence interval (CI); 0.98 (0.96,1.00), *p* value = 0.039). The highest quartile of dietary fiber intake exhibited a lower risk of gallstone disease in comparison with the lowest quartile (quartile 4 vs. quartile 1: 0.65 (0.45, 0.94), *p* value = 0.022). The inverse correlation between dietary fiber intake and the prevalence of gallstones was found to be statistically significant in several subgroups, including males, Hispanic individuals, those with less than a high school education, alcohol consumers, and individuals diagnosed with diabetes. Furthermore, the analysis of dose–response curves indicated a nearly linear correlation between dietary fiber intake and the risk of gallstone development.

**Conclusion:**

Dietary fiber intake is inversely associated with gallstone disease in United States adults. Adequate dietary fiber intake may be beneficial in reducing gallstone prevalence.

## Introduction

Gallstone disease, primarily composed of solidified cholesterol and bile pigments, is the most common digestive disorder necessitating hospitalization in Western countries (1, 2). The majority of individuals afflicted with gallstones do not manifest any symptoms, with a mere 2–4% of cases resulting in severe symptoms such as acute abdominal pain (3). Beyond typical presentations, gallstones rarely cause mechanical obstruction syndromes, including Bouveret syndrome (gastric outlet obstruction due to impacted stones) or gallstone ileus (small bowel obstruction secondary to stone migration) (4). Globally, the prevalence of gallstones has been documented to be approximately 6% in the 21st century (5). An epidemiological study of the United States population found that the prevalence of cholelithiasis increased from 7.4 to 13.9% over a period of nearly 30 years from 1988 to 2020 (6). This significant increase underscores the imperative for effective prevention strategies, as the burden that gallstones impose on healthcare systems is considerable (7). A rational and holistic diet has been demonstrated to be one of the most efficacious measures for the prevention of gallstone disease (8, 9).

Dietary fiber is a pivotal nutrient in the prevention of various chronic diseases, with the potential to regulate gut microbial ecology and metabolism, thereby impacting human health (10–12). Nevertheless, the correlation between dietary fiber intake and gallstone formation remains to be extensively studied. A recently conducted case–control study, incorporating a sample of 531 participants, has revealed a correlation between dietary fiber intake and the risk of gallstone formation (13). However, the correlation between dietary fiber intake and gallstones among the adult population of the United States remains unclear. Furthermore, extant investigations have exclusively concentrated on diminutive cohorts of a few hundred individuals, consequently lacking substantial, large-scale data to substantiate their findings. Additionally, the dose–response relationship between dietary fiber intake and gallstone risk remains uncertain. In light of the observed study gaps, we hypothesized that there exists a correlation and a dose–response trend between dietary fiber intake and gallstones in the United States population.

This study utilized the NHANES database, a nationally representative, large-scale, continually updated dataset, for a comprehensive analysis of the cross-sectional association between dietary fiber intake and gallstone prevalence. The findings were further delineated through stratified analyses, subgroup analyses, and restricted cubic spline analyses, which yielded more nuanced associations between dietary fiber intake and gallstones.

## Methods

### Study design

**Figure 1:**
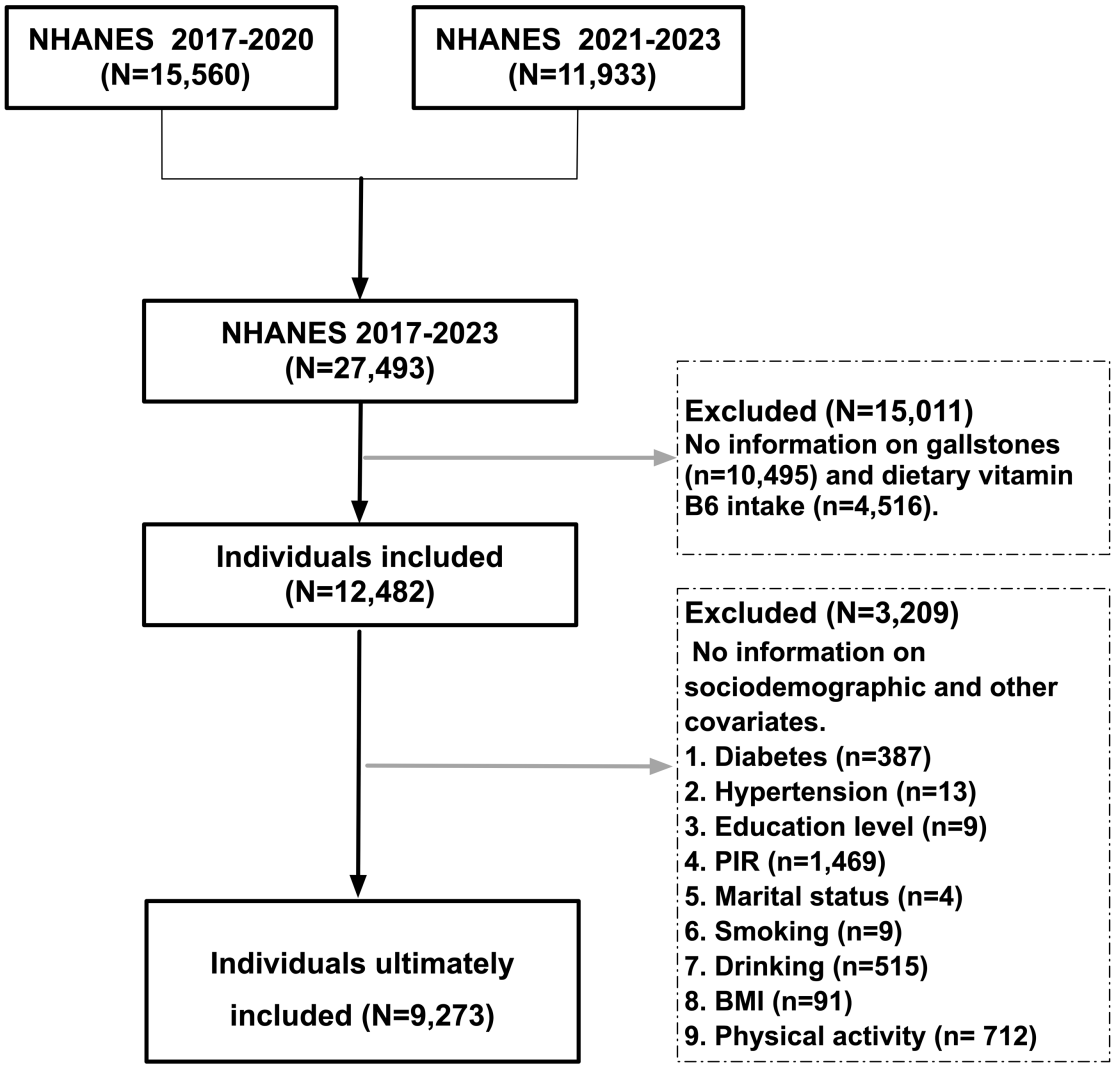
Screening flowchart for patient participation in the study. Following the integration of the total number of participants from the three cycles (2017–2023), a total of 9,273 participants with complete information on gallstones, dietary fiber, and important covariates were finally screened.

The National Health and Nutrition Examination Survey (NHANES) is a representative, large-scale, and ongoing public database that collects data on the health of adults and children in the United States (14). The database is updated on a biennial cycle, with approximately 5,000 adults and children in communities across the United States participating in NHANES annually (15, 16). The study was conducted in accordance with the Strengthening the Reporting of Observational Studies in Epidemiology (STROBE) statement guidelines, which are designed to standardize the presentation of findings in the field of epidemiology (17). The data set under consideration encompasses three cycles from 2017 to 2023. Participants were included if they met the following criteria: Firstly, participants were required to provide detailed information on their dietary fiber intake; Secondly, clear outcome information on gallstone disease and complete information on the corresponding covariates were expected. The detailed NHANES population screening process is illustrated in Figure 1.

### Dietary fiber intake and assessment of gallstone disease

Dietary fiber intake was quantified using two 24-h dietary recalls collected via in-person interviews by trained NHANES staff. The first recall was conducted in person at the Mobile Examination Center; the second was collected telephonically 3–10 days later.

Nutrient values—including total dietary fiber (grams/day)—were calculated using the USDA Food and Nutrient Database for Dietary Studies (FNDDS) and NHANES-specific food codes. Individual food items reported in each 24-h recall were matched to corresponding FNDDS entries, and their fiber content was summed to derive daily intake. The average fiber intake across both recalls was used as the representative value for each participant. No proprietary scoring system was applied; ‘scores’ in Table 1 refer to: Continuous fiber intake (g/day); Standardized intake (per 1 SD = 9.40 g); Quartiles (Q1–Q4) based on population-level distribution. This approach aligns with established NHANES nutrition analysis protocols (18). The diagnosis of gallstone disease is determined by the question, “Has the doctor ever said you have gallstones?” (19).

**Table 1:** ORs and 95%CIs of the association between dietary fiber intake and gallstones.

Dietary fiber intake (g)	^a^Model 1		^b^Model 2		^c^Model 3	
	OR 95%CI	*p* value	OR 95%CI	*p* value	OR 95%CI	*p* value
Fiber intake (continuous)	0.97 (0.96, 0.98)	**<0.001**	0.98 (0.96, 0.99)	**0.003**	0.98 (0.96, 1.00)	**0.039**
Per 1 SD (SD = 9.40)	0.77 (0.68, 0.86)	**<0.001**	0.81 (0.71, 0.93)	**0.003**	0.84 (0.71, 0.99)	**0.039**
Quartile of fiber intake						
7.40 Q1 [0, 10.05)	Ref.		Ref.		Ref.	
12.30 Q2 [10.05, 14.75)	0.81 (0.61, 1.06)	0.125	0.79 (0.59, 1.06)	0.115	0.82 (0.60, 1.13)	0.225
17.30 Q3 [14.75, 20.7)	0.75 (0.57, 0.99)	**0.040**	0.75 (0.56, 1.01)	0.059	0.80 (0.58, 1.11)	0.189
26.25 Q4 [20.7, 117.3]	0.51 (0.38, 0.67)	**<0.001**	0.59 (0.43, 0.81)	**0.001**	0.65 (0.45, 0.94)	**0.022**
*P* for trend	**<0.001**		**0.001**		**0.030**	

Bold values indicate statistical significance.

^a^Model 1 was the crude Model.

^b^Model 2 was adjusted for age, gender, race, education, PIR, and marital status.

^c^Model 3 represented a refinement of Model 2, incorporating additional adjustments for total energy, water intake, smoker, drinker, BMI, physical activity, diabetes, and hypertension.

OR, odds ratio; CI, confidence interval.

### Information on covariates

Information on potential confounding variables was selected with reference to previous studies and included age, gender, race, education, poverty-to-income ratio (PIR), marital status, total energy, water intake, smoking, drinking, BMI, physical activity, diabetes, and hypertension (20–24). Specifically, the data pertaining to race was divided into four categories: Hispanic, Non-Hispanic White, Non-Hispanic Black, and Other; education was classified into three categories: “less than high school education (<HS)”, “high school diploma (HS)”, and “college or above”; PIR was classified into three categories depending on what had been reported: 1. “PIR < 1”, 2. “1 to 4”, 3. “PIR > 4” (25); marital status was recorded according to the original NHANES data classification as 1. “married/living with partner”, 2. “widowed/divorced/separated”, and 3. “never married”; physical activity was graded as “sedentary,” “moderate,” and “vigorous”; diabetes was determined by a combination of whether or not one was taking insulin and a doctor’s diagnosis; hypertension was determined by a combination of whether or not one was taking antihypertensive medication and a doctor’s diagnosis. Furthermore, age was grouped into three categories in the stratified analyses: 1.20 to 39, 2.40 to 59, 3.60 +; BMI was further categorized as: 1. “underweight” (BMI < 18.5); 2. “normal” (18.5 ≤ BMI < 25.0); 3. “overweight” (25.0 ≤ BMI < 30.0); and 4. “obese” (BMI ≥ 30) (26).

### Statistical analysis

Continuous and categorical variables are presented as medians (interquartile range) and frequencies (percentage). The standardization of dietary fiber was conducted to formulate a Per 1 SD, and the quartile method was employed to present baseline patient information. A subsequent analysis of the differences in dietary fiber intake between patients without stones and those with stones was stratified by available covariates. An investigation was conducted to determine the correlation between dietary fiber intake and gallstones. To this end, three logistic regression models were constructed, and outcomes were presented as odd ratios (OR) and 95% confidence interval (CI): an unadjusted model 1, a model 2 adjusted for sociodemographic variables (age, gender, ethnicity, education, PIR, and marriage), and a model 3 further adjusted for health and lifestyle variables based on model 2 (total energy, water intake, smoker, drinker, BMI, physical activity, diabetes, and hypertension). It was also the case that subgroup and interaction analyses were performed and statistically significant subgroup results were presented. Finally, and as a point of particular significance, a restricted cubic spline (RCS) was utilized to simulate a dose–response curve between the ingestion of dietary fiber and the risk of gallstone prevalence. RCS used knots at the 5th, 35th, 65th, and 95th percentiles of dietary fiber intake. The implementation of all statistical methods was conducted utilizing SPSS 29.0 (International Business Machines, Armonk, NY, USA), Stata/MP 18.0 (Stata Corp, College Station, TX, USA) and R version 4.4.2 (R Core Team, Vienna, Austria). Results were deemed to be statistically significant if a two-sided *p* value less than 0.05 was exhibited.

## Results

### Description of population baseline

**Figure 2:**
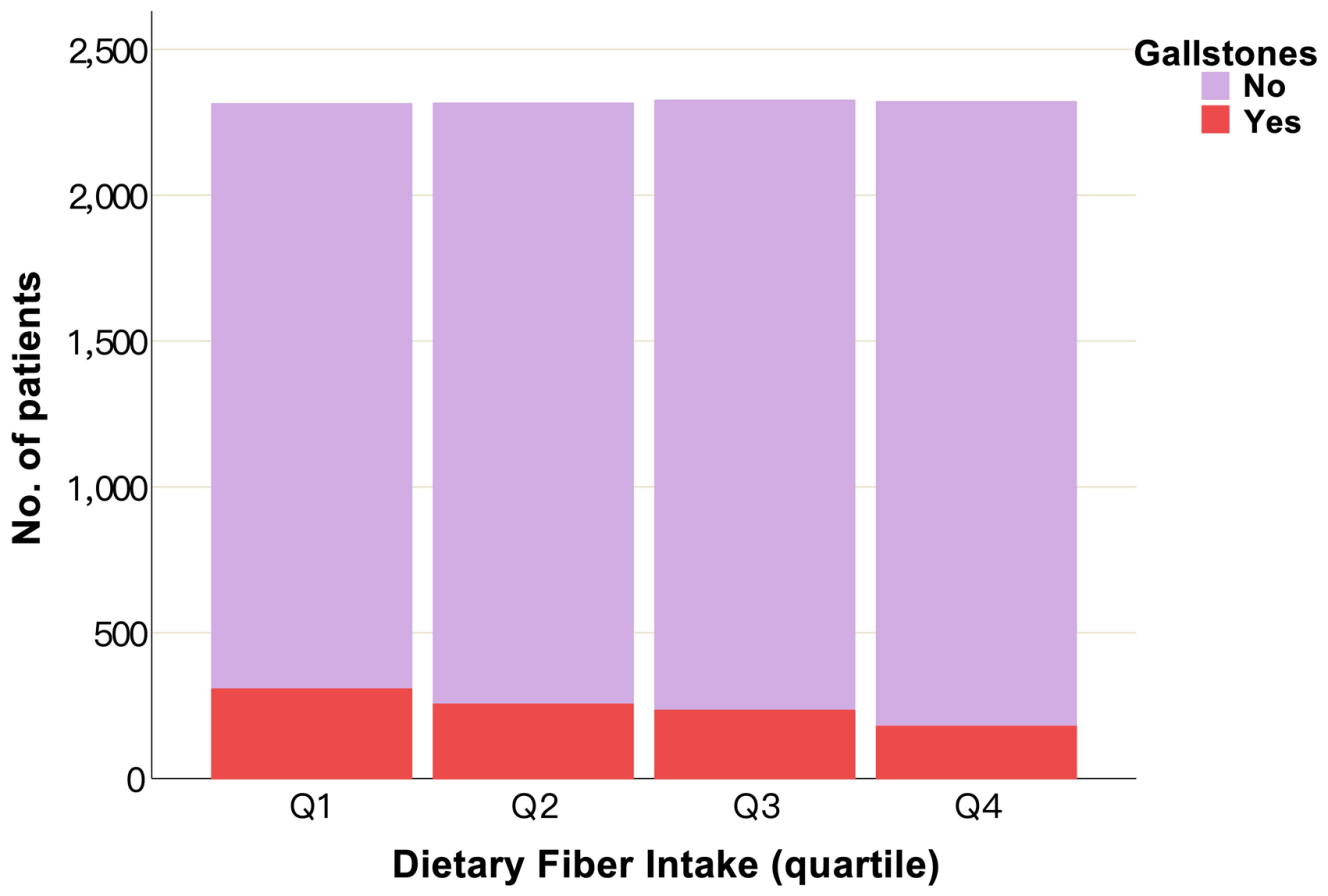
Histogram of the number of patients with and without gallstones according to dietary fiber quartiles. The height of the purple bar is indicative of the number of patients without gallstones, while the red color indicates the number of patients with gallstones. Q, quartile; No., number.

A total of 9,273 subjects were enrolled in the study, of which 971 had gallstones, yielding a prevalence of 10.47% (971/9,273). In comparison with the lowest quartile (Q1), patients with the highest quartile (Q4) of dietary fiber intake exhibited lower BMI (27 (24, 32) vs. 30 (25, 35)), proportion of women (39% vs. 60%), proportion of smokers (39% vs. 48%), proportion of individuals with a sedentary lifestyle (16% vs. 24%), proportion of individuals with diabetes (10% vs. 13%), and proportion of individuals with hypertension (27% vs. 35%); and higher energy intake (2,584 (2,040, 3,208) vs. 1,420 (1,123, 1,801)), water intake (3,364 (2,682, 4,211) vs. 1,420 (1,123, 1,801)), PIR (3.87 (1.99, 5.00) vs. 2.65 (1.31, 4.81)), proportion of individuals with “college or above” education (75% vs. 54%), proportion of individuals with “married/living with partner” status (67% vs. 55%), and proportion of individuals engaging vigorous physical activity (38% vs. 32%) (Table 2). Higher dietary fiber consumption was associated with a reduced prevalence of gallstones across quartiles (Q1, 13%; Q2, 11%; Q3, 9.9%; Q4, 6.9%; Table 2; Figure 2). Baseline characteristics stratified by gallstone status are presented in Supplementary Table S1.

**Table 2:** Basic information grouped by dietary fiber quartiles.

Characteristic	Q1, *N* = 2313^1^ (≥ 0, < 10.05)	Q2, *N* = 2315^1^ (≥14.75, < 20.70)	Q3, *N* = 2325^1^ (≥14.75, < 20.70)	Q4, *N* = 2320^1^ (≥20.70, < 117.3)	*p*-value^3^
Age (years)^2^	46 (31, 60)	47 (32, 62)	50 (34, 63)	47 (34, 61)	0.008
BMI (kg/m2)	30 (25, 35)	29 (25, 34)	28 (25, 33)	27 (24, 32)	<0.001
Total energy intake (kcal)	1,420 (1,123, 1801)	1835 (1,496, 2,273)	2,124 (1734, 2,623)	2,584 (2040, 3,208)	<0.001
Water intake (g)	2,213 (1,626, 3,025)	2,560 (1971, 3,354)	2,822 (2,213, 3,672)	3,364 (2,682, 4,211)	<0.001
PIR	2.65 (1.31, 4.81)	3.30 (1.71, 5.00)	3.61 (2.06, 5.00)	3.87 (1.99, 5.00)	<0.001
Female, *n* (%)	1,411 (60%)	1,298 (56%)	1,202 (49%)	956 (39%)	<0.001
Race					<0.001
Hispanic	346 (13%)	374 (13%)	450 (14%)	547 (19%)	
Non-Hispanic White	997 (64%)	1,144 (68%)	1,166 (68%)	1,062 (63%)	
Non-Hispanic Black	677 (15%)	530 (12%)	394 (8.4%)	308 (6.8%)	
Others	293 (8.7%)	267 (7.9%)	315 (9.5%)	403 (12%)	
Education					<0.001
< HS	405 (12%)	287 (8.0%)	236 (5.8%)	322 (8.9%)	
HS diploma	703 (35%)	540 (27%)	441 (23%)	351 (16%)	
College or above	1,205 (54%)	1,488 (65%)	1,648 (71%)	1,647 (75%)	
Married/Living with partner	1,145 (55%)	1,304 (63%)	1,404 (65%)	1,501 (67%)	<0.001
Smoker	1,100 (48%)	969 (41%)	891 (37%)	894 (39%)	<0.001
Drinker	2,105 (93%)	2,155 (94%)	2,139 (94%)	2,131 (92%)	0.2
Physical activity					0.002
Sedentary	675 (24%)	562 (20%)	522 (19%)	456 (16%)	
Moderate	1,013 (44%)	1,085 (48%)	1,130 (47%)	1,129 (46%)	
Vigorous	625 (32%)	668 (33%)	673 (34%)	735 (38%)	
Gallstones, yes	306 (13%)	254 (11%)	233 (9.9%)	178 (6.9%)	0.001
Diabetes, yes	370 (13%)	327 (11%)	324 (11%)	314 (10%)	0.3
Hypertension, yes	939 (35%)	860 (31%)	860 (31%)	750 (27%)	<0.001

Higher dietary fiber consumption was associated with a reduced prevalence of gallstones across quartiles (Q1, 13%; Q2, 11%; Q3, 9.9%; Q4, 6.9%).^1^N not Missing (unweighted); ^2^Median (Q1, Q3); n (unweighted) (%); ^3^Design-based Kruskal Wallis test; Pearson’s X^2: Rao & Scott adjustment. PIR, ratio of family income to poverty; BMI, body mass index; HS, high school.

### A comparison of dietary fiber intake between individuals afflicted with gallstones and those without gallstones

**Figure 3:**
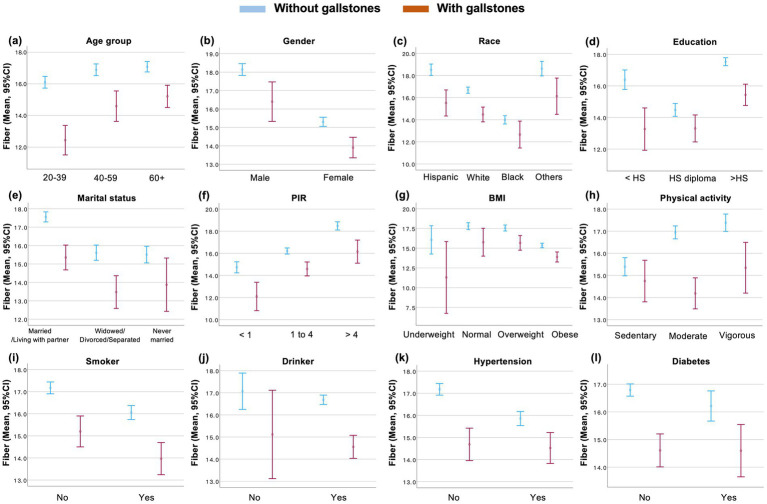
Dietary fiber intake in patients with and without gallstones stratified by covariates. **(a–l)** Dietary fiber intake was presented according to different covariates and gallstones. The center dot is used to denote the intake of dietary fiber, with the lines above and below the dot representing the error bars. PIR, ratio of family income to poverty; BMI, body mass index; HS, high school. CI, confidence interval.

In order to conduct a more in-depth investigation into the disparities observed in dietary fiber intake between patients with and without gallstones, a series of stratified analyses were performed. The results demonstrated that, in comparison with individuals not afflicted with gallstone disease, those diagnosed with gallstone disease exhibited a lower intake of dietary fiber, with this discrepancy being evident across various demographic and health-related strata, including age, gender, race, educational attainment, marital status, PIR, BMI, physical activity, smoking, drinking, hypertension, and diabetes (Figure 3). Furthermore, the disparity in dietary intake between patients with gallstone disease and those without was more pronounced in specific demographic groups, including adults aged 20 to 39 years, males, Hispanic individuals, those with less than high school education, PIR < 1, underweight, moderate intensity physical activity, non-hypertensive, and non-diabetic (Figure 3).

### The association of dietary fiber intake with gallstone disease in United States adults

**Figure 4:**
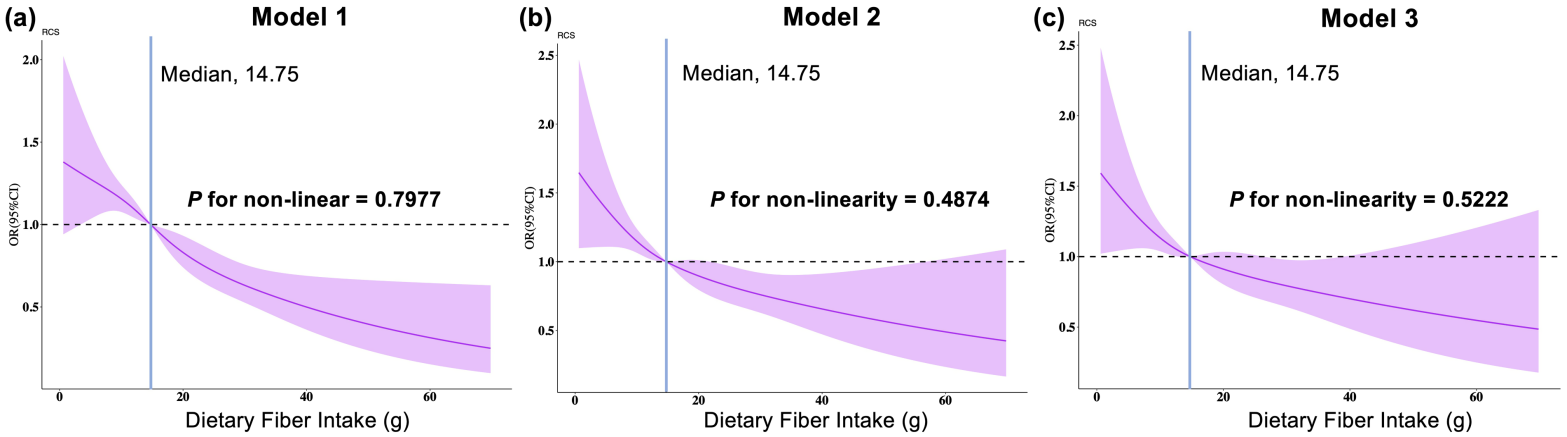
Restricted cubic spline for the relationship between dietary fiber intake and gallstones. **(a)** Model 1 was the crude model. **(b)** Model 2 was adjusted for age, gender, race, education, PIR, and marital status. **(c)** Mode3 was the fully adjusted model incorporating additional adjustments for total energy, water intake, smoker, drinker, BMI, physical activity, diabetes, and hypertension. OR, odds ratio; CI, confidence interval.

In the subsequent phase of the study, three models were adapted in order to investigate the association between dietary fiber intake and gallstone disease. In the final adjusted model 3, a inverse correlation was identified between dietary fiber intake and gallstones (odds ratio (95% confidence interval, 0.98 (0.96, 1.00), *p* = 0.039)). The inverse correlation was further substantiated by logistic regression analyses for standardized dietary fiber intake (per 1 SD, 0.84(0.71, 0.99), *p* = 0.039) and quartiles (Q4 vs. Q1, 0.65(0.45, 0.94), *p* = 0.022, *P* for trend = 0.030) (Table 1). Additionally, a dose–response curve was simulated between dietary fiber intake and the risk of gallstones utilizing an RCS. The results of the RCS demonstrated that an elevated dietary fiber intake was associated with a reduced risk of gallstones and that the inverse correlation between dietary fiber intake and gallstones exhibited an approximate linear relationship (Figure 4).

### Subgroup and interaction analysis

To ascertain which population demonstrated a high degree of sensitivity to the relationship between dietary fiber intake and gallstone disease, subgroup and interaction analyses were performed. The results of the subgroup analyses indicated a statistically significant inverse correlation between dietary fiber intake and gallstone disease in the following populations: males (OR (95%CI), 0.96 (0.93, 0.99)), Hispanic individuals (0.95 (0.92, 0.98)), those with less than a high school diploma (<HS) in education (0.94 (0.91, 0.98)), alcohol drinkers (0.98 (0.96, 1.00)), and individuals with diabetes (0.97 (0.93, 1.00)) (Table 3). Interaction analyses detected no variables that significantly altered the relationship between dietary fiber intake and gallstone disease (Table 3).

**Table 3:** The relationship between dietary fiber intake and gallstones, stratified by gender, race, education, drinker and diabetes.

Stratified variable		OR 95CI%	*P* for interaction
All patients		**0.98 (0.96, 1.00)**	
Gender	Male	**0.96 (0.93, 0.99)**	0.386
	Female	0.99 (0.97, 1.01)	
Race	Hispanic	**0.95 (0.92, 0.98)**	0.961
	Non-Hispanic White	0.99 (0.97, 1.01)	
	Non-Hispanic Black	0.98 (0.95, 1.01)	
	Other	0.98 (0.95, 1.00)	
Education	< HS	**0.94 (0.91, 0.98)**	0.488
	HS diploma	0.98 (0.94, 1.02)	
	College or above	0.99 (0.97, 1.01)	
Drinker	Yes	**0.98 (0.96, 1.00)**	0.485
	No	1.00 (0.96, 1.04)	
Diabetes	Yes	**0.97 (0.93, 1.00)**	0.973
	No	0.98 (0.97, 1.00)	

A fully adjusted model was employed in this analysis, adjusting for age, gender, race, education, PIR, marital status, total energy, water intake, smoker, drinker, BMI, physical activity, diabetes, and hypertension. OR, odds ratio; CI, confidence interval.Bold values indicate statistical significance.

## Discussion

In this study, we analyzed the most recent NHANES data from 2017 to 2023, and for the first time, we found a nearly linear inverse association between dietary fiber intake and gallstones among United States adults. A significant discrepancy was also observed in the dietary fiber intake of patients with gallstones in comparison to those without gallstones. The inverse correlation between dietary fiber intake and gallstones was found to be statistically significant in specific subgroups, including men, Hispanic individuals, those with less than a high school diploma, individuals with alcohol consumption, and those with a diagnosed history of diabetes.

This study addressed a significant research gap in the extant literature on this subject, as it is the first to examine the relationship between dietary fiber and gallstone formation in a population-based sample in the United States. The primary conclusion of the study was that for every additional 1 g of dietary fiber consumed, there was a 2% reduction in the risk of gallstone prevalence. This finding aligns with previous research that has also demonstrated a inverse correlation between the intake of dietary fiber (both soluble and insoluble) and the prevalence of gallstones (13). The antioxidant activity of dietary fiber is a globally recognized phenomenon (27–29). A significant benefit of the antioxidant-rich, anti-inflammatory diet that has been demonstrated in a multitude of studies is its potential to contribute to a reduction in the prevalence of gallstones among American adults (30–32). It is noteworthy that ultra-processed foods frequently exhibit a paucity of dietary fiber and have recently been demonstrated to be associated with unfavorable gallstone outcomes (33–35). The preceding findings, whether primary or secondary in nature, provide direct or indirect confirmation of the preventive effect of adequate dietary fiber intake on gallstone disease, thereby providing substantial support for the primary findings of this study.

In subgroup analyses, the sensitive populations for the relationship between dietary fiber and gallstones were identified as men, Hispanics, those with less than high school education, alcohol consumers, and diabetics. The persistent identification of women as a risk factor for gallstones may obscure the protective effect of dietary fiber on gallstones in women (2, 36). Consequently, it is plausible that the relationship between dietary fiber and gallstones is only significant in men. The NHANES survey demonstrated that Hispanics exhibited a higher prevalence of gallstones compared to non-Hispanics (205/1717 (11.94%) vs. 766/7556 (10.14)), suggesting that environmental, cultural, dietary, and genetic factors contribute to the variation in gallstone prevalence (37, 38). This observation lends further support to the hypothesis that these factors may play a role in the increased sensitivity to gallstones observed in Hispanic populations. Furthermore, a correlation has been observed between diabetes, alcohol consumption, and gallstone formation, indicating that individuals with diabetes and those who consume alcohol are more likely to benefit from a diet high in fiber (24, 39–41). It is evident that there is an absence of substantial evidence to substantiate the internal logic of the identification of these sensitive populations. The subgroup findings (e.g., Hispanic individuals) should be interpreted as hypothesis-generating due to potential type I error from multiple testing. Large cohort studies or epidemiological research in specific populations are imperative to validate our findings.

Despite the absence of a definitive understanding of the underlying mechanisms, previous research reports offer valuable insights into the potential impact of dietary fiber intake on gallstone disease. A primary rationale for this phenomenon pertains to the protective function of dietary fiber, which promotes peristalsis within the gastrointestinal tract and enhances intestinal flora (42–44). The improvement of human gut bacteria has been demonstrated to have a positive effect on the biotransformation of bile salts, thus effectively preventing gallstones at source (45–47). Conversely, the proliferation of specific detrimental intestinal microbiota (especially *Desulfovibrionales*) has also been observed to promote the development of gallstones (48). It has been ascertained that dietary fiber is subject to fermentation by the gut microbiota present within the colon, thereby yielding significant quantities of short-chain fatty acids (e.g., acetate, propionate, and butyrate) (49). These acid metabolites represent pivotal intermediate stages in the body’s more profound metabolism and have the capacity to regulate bile acid metabolism by modulating the gut microbiota. For instance, sodium butyrate has been demonstrated to modulate bile acid metabolism in conjunction with intestinal microflora, thus providing a potential therapeutic intervention for cholesterol gallstones (50). To summarize, the examination of the preventative mechanisms of dietary fiber in relation to the development of gallstones and its impact on the underlying pathophysiology remains in its initial stages. In order to substantiate these findings, it is imperative that large-scale randomized controlled trials and in-depth fundamental mechanistic studies are conducted in the future.

However, it is important to note that the study was subject to certain limitations. Firstly, the reports of dietary fiber intake and gallstone disease were derived from self-recall and may be subject to bias. Secondly, the cross-sectional design inherently limits causal inference; our findings demonstrate an association rather than establishing causality. Thirdly, although we adjusted for major known confounders (including diabetes, hypertension, BMI, and lifestyle factors), we acknowledge the absence of data on dyslipidemia (e.g., serum LDL/HDL cholesterol), hypertriglyceridemia, hematological disorders, and detailed dietary patterns (e.g., ketogenic, high-fat, or low-carbohydrate diets) in our models. These factors may influence gallstone pathogenesis through bile cholesterol saturation, hemolytic effects on bilirubin metabolism, or dietary interactions with fiber. While NHANES provides triglyceride measurements for subsets, comprehensive dyslipidemia diagnosis and hematologic disease data were unavailable for our full cohort across cycles. Our study did not evaluate holistic dietary patterns (e.g., Western diets) due to the nutrient-specific focus on fiber. Future studies incorporating these clinical variables would strengthen causal inference.

## Conclusion

In summary, the present study demonstrated, for the first time, an inverse correlation between dietary fiber intake and gallstone disease in an adult population in the United States. However, residual confounding and the observational design preclude definitive causal conclusions. Large-scale multi-center clinical trials and epidemiological studies are imperative to validate this finding. Ensuring adequate dietary fiber intake may be a plausible initiative to protect patients from gallstones. Specifically, whole grains with high levels of dietary fiber (such as oatmeal or oats), nuts and seeds (such as chia seeds or flax seeds), and cruciferous vegetables (including broccoli or Brussels sprouts) are worthy of consideration.

## Data Availability

Publicly available datasets were analyzed in this study. This data can be found here: https://wwwn.cdc.gov/nchs/nhanes/default.aspx.
