# Supplementary material for: Inverse association between dietary fiber intake and gallstone disease in U.S. adults: a cross-sectional study from the NHANES database
Source: Front Nutr. 2025 Jul 1;12:1624173. doi: 10.3389/fnut.2025.1624173 (PMC12259441; doi:10.3389/fnut.2025.1624173)
Supplement: Supplementary file 1 [file Data_Sheet_1.zip › Suppl.Table S1/Table S1.docx]

**Table S1**. Baseline characteristics stratified by gallstone status.

| **Characteristic** | **Overall,**  **N = 9273^1^** | **Group** **by gallstone status** | | ***P*-value**^3^ |
| --- | --- | --- | --- | --- |
|  |  | **Without gallstone,**  **N = 8302^1^** | **With gallstone,**  **N = 971^1^** |  |
| **Age (years) ^2^** | 47 (33, 62) | 46 (32, 60) | 59 (46, 69) | **<0.001** |
| **BMI (kg/m2)** | 29 (25, 33) | 28 (24, 33) | 31 (27, 38) | **<0.001** |
| **Fiber (g)** | 15 (10, 21) | 15 (10, 21) | 13 (9, 18) | **<0.001** |
| **Total energy intake (kcal)** | 1,962 (1,511, 2,543) | 1,982 (1,531, 2,572) | 1,766 (1,391, 2,243) | **<0.001** |
| **Water intake (g)** | 2,756 (2,076, 3,619) | 2,774 (2,083, 3,629) | 2,620 (2,017, 3,448) | **0.036** |
| **PIR** | 3.37 (1.74, 5.00) | 3.42 (1.77, 5.00) | 2.92 (1.58, 4.80) | **<0.001** |
| **Female, n (%)** | 4,867 (51%) | 4,168 (49%) | 699 (74%) | **<0.001** |
| **Race** |  |  |  | **0.026** |
| Hispanic | 1,717 (15%) | 1,512 (15%) | 205 (14%) |  |
| Non-Hispanic White | 4,369 (66%) | 3,866 (65%) | 503 (70%) |  |
| Non-Hispanic Black | 1,909 (10%) | 1,758 (11%) | 151 (6.8%) |  |
| Others | 1,278 (9.5%) | 1,166 (9.5%) | 112 (8.7%) |  |
| **Education** |  |  |  | **0.011** |
| < HS | 1,250 (8.5%) | 1,108 (8.4%) | 142 (9.5%) |  |
| HS diploma | 2,035 (25%) | 1,801 (25%) | 234 (30%) |  |
| College or above | 5,988 (66%) | 5,393 (67%) | 595 (60%) |  |
| **Married/Living with partner** | 5,354 (63%) | 4,797 (63%) | 557 (62%) | **<0.001** |
| **Smoker** | 3,854 (41%) | 3,384 (40%) | 470 (51%) | **<0.001** |
| **Drinker** | 8,530 (93%) | 7,641 (93%) | 889 (93%) | 0.7 |
| **Physical activity** |  |  |  | **0.001** |
| Sedentary | 2,215 (19%) | 1,935 (19%) | 280 (25%) |  |
| Moderate | 4,357 (46%) | 3,880 (46%) | 477 (47%) |  |
| Vigorous | 2,701 (34%) | 2,487 (35%) | 214 (27%) |  |
| **Diabetes, yes** | 1,335 (11%) | 1,087 (10%) | 248 (21%) | **<0.001** |
| **Hypertension, yes** | 3,409 (31%) | 2,905 (29%) | 504 (48%) | **<0.001** |

Group comparisons accounted for NHANES sampling weights. ^1^N not Missing (unweighted); ^2^Median (Q1, Q3); n (unweighted) (%); ^3^Design-based Kruskal Wallis test; Pearson's X^2: Rao & Scott adjustment. PIR, ratio of family income to poverty; BMI, body mass index; HS, high school.
